# Acute Pancreatitis as a Rare Initial Presentation of Plasma Cell Leukemia in a Young Male: A Case Report

**DOI:** 10.7759/cureus.111117

**Published:** 2026-06-18

**Authors:** Pritha Chatterjee, Anirban Kundu, Sulagna Giri, Atoshi Basu

**Affiliations:** 1 Pathology and Laboratory Medicine, Apollo Multispeciality Hospitals, Kolkata, IND; 2 Hematopathology, Apollo Multispeciality Hospitals, Kolkata, IND

**Keywords:** acute pancreatitis, cytogenetics, extra medullary myeloma, flow cytometry, plasma cell leukemia

## Abstract

Plasma cell leukemia (PCL) is a rare and aggressive plasma cell neoplasm characterized by the presence of neoplastic plasma cells in the peripheral blood. It may arise de novo (primary PCL) or may evolve from a previously existing plasma cell neoplasm (secondary PCL). It is typically a disease of old age, with a median age of incidence of approximately 55 years. Primary PCL is often considered a manifestation of extra-medullary myeloma (EMM) and is typically associated with extensive organ involvement, while lacking extensive osteolytic lesions. We present the case of a 24-year-old male patient who presented with acute pancreatitis without an initial suspicion of a hematological malignancy and whose routine peripheral blood evaluation, followed by bone marrow examination and flow cytometry, revealed a diagnosis of primary PCL.

## Introduction

Plasma cell leukemia (PCL) is a rare and aggressive hematological malignancy of clonal plasma cells characterized by involvement of the peripheral blood. First described more than a century ago, the diagnosis and treatment of PCL have undergone considerable changes. It may present de novo as primary PCL (pPCL) or may evolve from a pre-existing plasma cell neoplasm as secondary PCL [1,2].

Traditionally, the diagnosis of PCL was based on the presence of >20% circulating plasma cells or an absolute plasma cell count >2 × 10⁹/L in peripheral blood [3]. However, the revised International Myeloma Working Group (IMWG) diagnostic criteria in 2021 define PCL by the presence of ≥5% circulating plasma cells in peripheral blood in patients with symptomatic multiple myeloma (MM), as this threshold was found to have similar adverse prognostic significance [4].

It has a lower median age at presentation (52 to 65 years) in comparison to MM, and may have a varied clinical presentation with a spectrum of clinical manifestations, including anemia, thrombocytopenia, recurrent infections, hepatosplenomegaly, renal dysfunction, hypercalcemia, and extra-medullary involvement [1,2]. While MM typically presents with CRAB features (hypercalcemia, renal dysfunction, anemia and lytic bony lesions), the occurrence of hypercalcemia and bony lesions is relatively lower in pPCL. It has a significantly inferior prognosis compared to MM and prompt identification and treatment is essential [5].

Extra-medullary myeloma (EMM) occurs when plasma cells escape the bone marrow microenvironment and infiltrate soft tissues, distant organs, or para-osseous tissues [6]. pPCL is distinct from classical MM and shares a biological and molecular landscape overlapping with EMM, including increased genomic instability and reduced dependence on the bone marrow microenvironment [6,7]. Deep sequencing studies have identified a higher frequency of high-risk chromosomal abnormalities in EMM, including 1q21 amplification, which promotes plasma cell proliferation and survival through genes such as *CKS1B* and *BCL9*; *TP53* deletion, which facilitates escape from the bone marrow microenvironment and independent extra-medullary growth; and del(1p32), which is more commonly associated with secondary EMM and disease progression. Several secondary and terminal genetic events in the natural disease course of myeloma, including high-risk genetic aberrations such as deletion 13q and deletion 17p, may also have a role in extra-medullary disease [6,8]. Immunophenotypic variations, such as loss of CD56 and other homing receptors, are commonly observed in EMM and PCL and play an important role in extra-medullary dissemination [7,9].

Even with the wide heterogeneity in clinical presentation of PCL, young age of presentation and acute pancreatitis as the initial presentation remains an uncommon occurrence and is usually related to malignancy-associated hypercalcemia [10-13]. In this case report, we describe a young adult presenting with acute pancreatitis as the initial manifestation of pPCL and also attempt to explain the underlying pathophysiology.

## Case presentation

A 24-year-old male patient presented with a five-day history of acute-onset epigastric pain associated with multiple episodes of vomiting. There was no history of alcohol consumption, gallstones, recent drug intake, trauma, or comorbid illness. On physical examination, the patient was febrile but hemodynamically stable. There was no pallor, icterus, lymphadenopathy, or hepatosplenomegaly. Abdominal examination revealed epigastric tenderness without guarding or rigidity.

The case was investigated comprehensively utilizing morphological examination of the peripheral blood and bone marrow, flow cytometry immunophenotyping, biochemical analysis, and fluorescence in situ hybridization (FISH).

Laboratory investigations revealed hypercalcemia (serum calcium: 13.2 mg/dL) with markedly elevated pancreatic enzymes (serum lipase: 1830 U/L; serum amylase: 658 U/L), consistent with acute pancreatitis. Additional findings included elevated total protein (12.3 g/dL) with hypoalbuminemia (2.8 g/dL), resulting in a reversed albumin/globulin ratio (0.3), and impaired renal function (serum creatinine: 2.4 mg/dL). Erythrocyte sedimentation rate (ESR) was markedly elevated (92 mm/hr). The complete blood count (CBC) revealed mild anemia (Hb 11.6 g/dL), severe thrombocytopenia (platelet count 21,000/µL), and leukocytosis (white blood cell count (WBC) 32,100/µL). The laboratory parameters along with their reference ranges have been tabulated below (Table 1).

**Table 1:** Laboratory parameters at presentation along with standard reference ranges

Parameter	Patient Value	Normal Reference range [14-16]
Hemoglobin (Hb)	11.6 g/dL	13.0–17.0 g/dL
Total Leucocyte Count (WBC)	32,100/µL	4,000–11,000/µL
Platelet Count	21,000/µL	150,000–450,000/µL
Erythrocyte sedimentation rate (ESR)	92 mm/hr	0–15 mm/hr
Serum Calcium	13.2 mg/dL	8.5–10.5 mg/dL
Serum Creatinine	2.4 mg/dL	0.7–1.3 mg/dL
Total Protein	12.3 g/dL	6.0–8.3 g/dL
Serum Albumin	2.8 g/dL	3.5–5.0 g/dL
Albumin/Globulin Ratio	0.3	1.0–2.2
Serum Lipase	1830 U/L	13–60 U/L
Serum Amylase	658 U/L	30–110 U/L

The peripheral blood smear demonstrated 51% atypical plasma cells (Figure 1).

**Figure 1:**
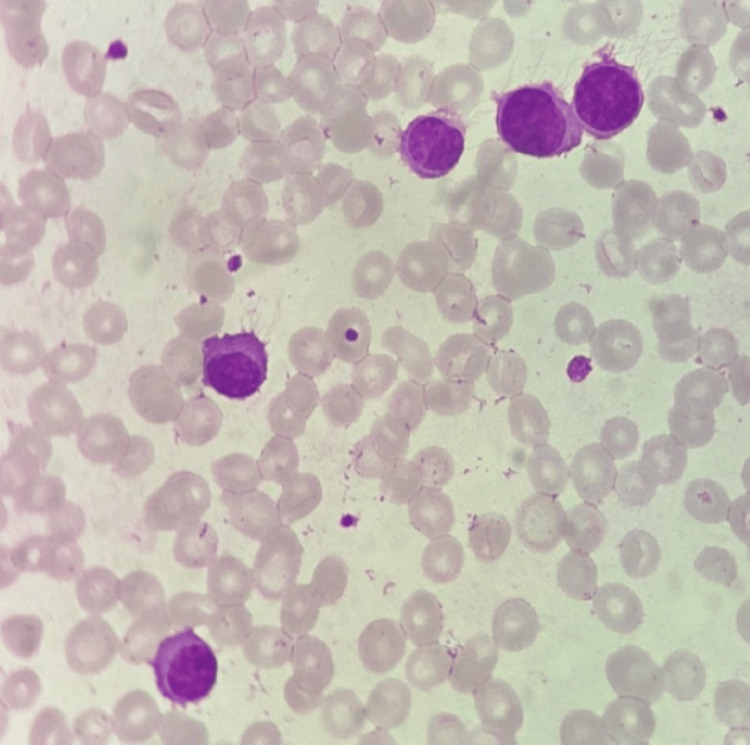
Peripheral smear demonstrating numerous plasma cells at 1000x magnification

Abdominal ultrasonography showed bulky, edematous pancreas. Contrast-enhanced computed tomography (CECT) of the abdomen confirmed features of necrotizing pancreatitis, without evidence of gallstones or biliary obstruction (Figures 2, 3).

**Figure 2:**
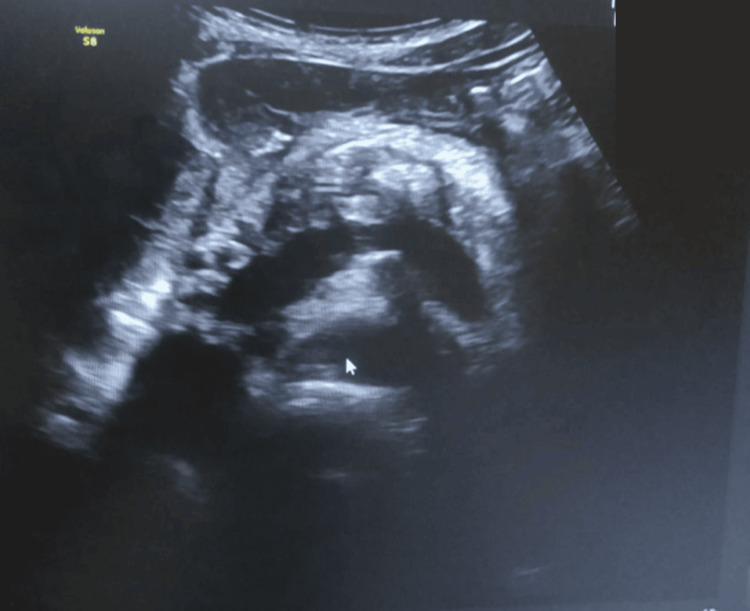
Transabdominal ultrasonography Shows a bulky pancreas with heterogeneous echotexture and indistinct margins along with mild peripancreatic inflammatory change/fluid adjacent to the gland; suggestive of acute pancreatitis.

**Figure 3:**
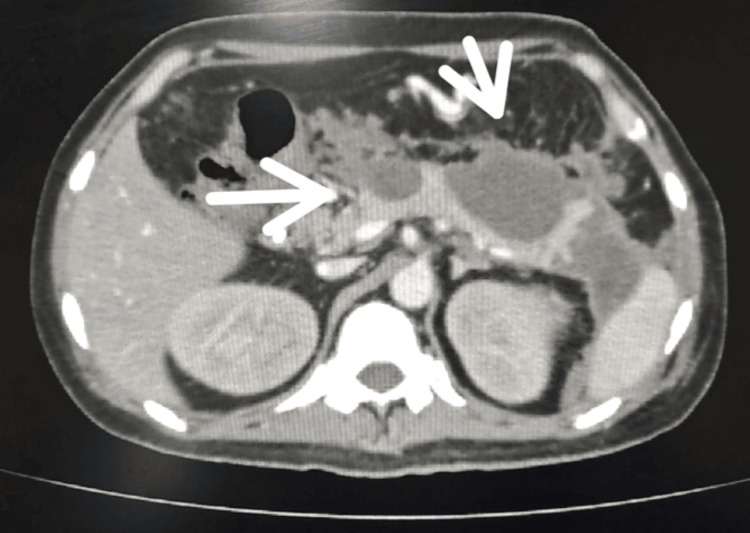
Contrast-enhanced computed tomography (CECT) abdomen in axial section Shows bulky edematous pancreas with peripancreatic inflammatory changes and fluid collection (marked by arrows); suggestive of acute necrotizing pancreatitis.

CT imaging of the thorax revealed a lytic lesion in the scapula (Figures 4A, 4B).

**Figure 4:**
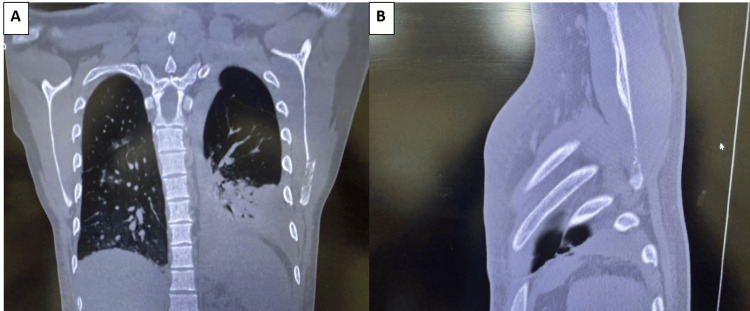
CT thorax showing left hydropneumothorax with adjacent left scapular erosion

Markedly elevated serum IgG levels (7680 mg/dL) and a prominent monoclonal (M) band (4.9 g/dL) on serum protein electrophoresis (SPEP) was noted (Figure 5 and Table 2).

**Figure 5:**
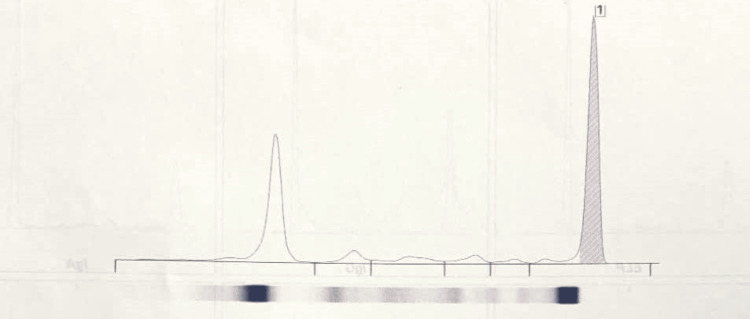
Serum protein electrophoresis (SPEP) showing presence of large M band

**Table 2:** Various fractions in serum protein electrophoresis (SPEP) with their standard reference ranges [17]

Fraction	%	Reference (%)	Concentration (g/dL)	Reference concentration (g/dL)
Albumin	35.7	55.8–66.1	3.8	4.0–4.8
Alpha-1	4.6	2.9–4.9	0.5	0.2–0.4
Alpha-2	5.3	7.1–11.8	0.6	0.5–0.9
Beta-1	3.5	4.7–7.2	0.4	0.6–0.9
Beta-2	1.7	3.2–6.5	0.2	0.2–0.5
Gamma	49.2	11.1–18.8	5.2	0.8–1.6

Beta-2 microglobulin was significantly elevated (31.4 mg/L). Serum free light chain assay showed lambda light chain restriction.

Bone marrow aspiration revealed 54% plasma cells, comprising of both mature and immature forms, while trephine biopsy showed a hypercellular marrow with diffuse sheets of abnormal plasma cells, with suppression of normal hematopoiesis. No definitive evidence of any plasmablastic differentiation was noted (Figures 6A, 6B).

**Figure 6:**
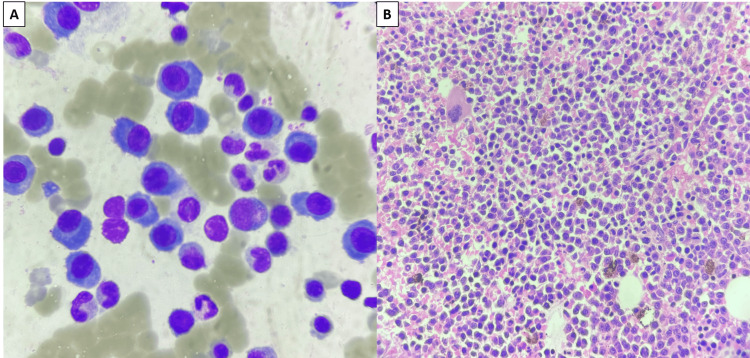
Bone marrow showing diffuse plasma cell infiltration A) Bone marrow aspirate (1000x magnification); B) Bone marrow biopsy (400x magnification).

Flow cytometry analysis performed on peripheral blood revealed 61.8% plasma cells on primary gating with CD138 and CD38. Approximately 50.5% cells were abnormal plasma cells showing negative to dim CD45 expression and negative CD19 expression. These cells showed Human Leukocyte Antigen - DR isotype (HLA-DR) positivity and lambda light chain restriction, along with negative CD56 expression, thus confirming a diagnosis of PCL (Figure 7).

**Figure 7:**
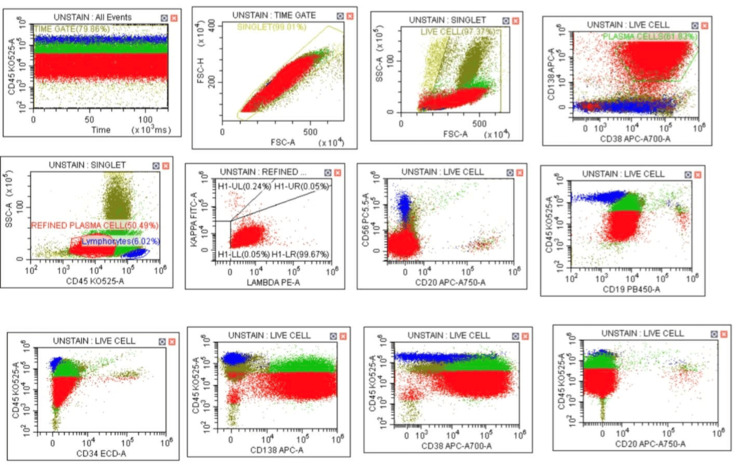
Flow cytometry immunophenotyping from peripheral blood shows approximately 50.5% clonal plasma cells with lambda light chain restriction

Subsequently, a myeloma FISH panel was performed for the patient which showed 1q amplification (Figure 8).

**Figure 8:**
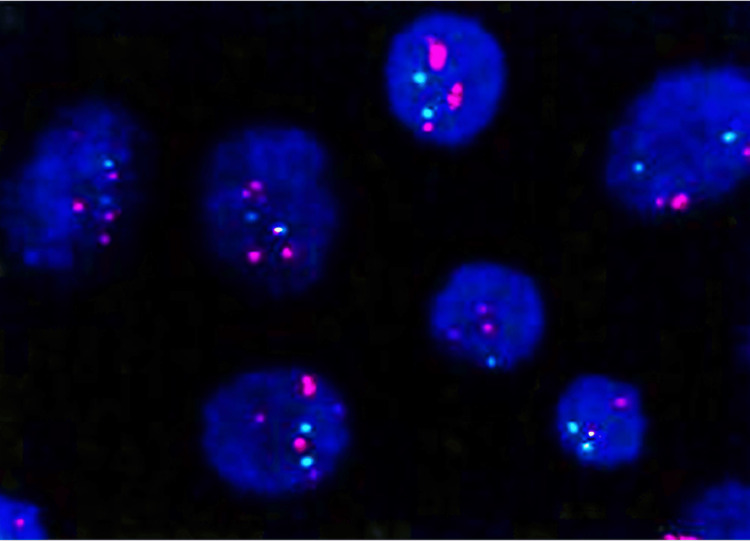
Fluorescence in situ hybridization (FISH) using Metasystem 1p32/1q21 deletion/amplification probes, demonstrating preferential gain of 1q21 (red signal)

The patient was initiated on combination chemotherapy comprising carfilzomib, lenalidomide, dexamethasone, and daratumumab. Following induction, he underwent autologous stem cell transplantation (ASCT). The patient demonstrated significant clinical and biochemical improvement, with resolution of pancreatitis and stabilization of systemic disease. Post ASCT, the patient was started on consolidation with bortezomib, pomalidomide, and dexamethasone and is doing well after completing six cycles of consolidation therapy.

## Discussion

PCL is a rare and aggressive plasma cell dyscrasia (PCD) characterized by circulating clonal plasma cells in peripheral blood. It may occur de novo (pPCL) or evolve from pre-existing MM (secondary PCL) [1,2]. Although the median age at diagnosis is generally lower than MM, our patient presented at the exceptionally young age of 24 years, making this presentation distinctly uncommon [1,2].

Recent IMWG recommendations emphasize that lower percentages of circulating plasma cells may carry prognostic significance and support the diagnosis of pPCL in the appropriate clinical setting [4]. Clinically, pPCL demonstrates a more aggressive biological behaviour than conventional MM, with higher tumor burden, extensive extra-medullary involvement, renal dysfunction, cytopenias, and elevated beta-2 microglobulin levels [5,18]. In contrast to classical MM, which commonly presents with osteolytic lesions and bone pain, pPCL frequently manifests with systemic and extra-medullary features due to hematogenous dissemination of plasma cells [6,7]. In our case, the patient presented initially with acute pancreatitis, a highly unusual manifestation that delayed suspicion of an underlying haematological malignancy.

Acute pancreatitis associated with MM or PCL is rare and can be attributed to severe hypercalcemia. To the best of our knowledge, no definitive data on pancreatitis as an initial manifestation of PCL exists; however, documented literature reports pancreatitis secondary to hypercalcemia of hyperparathyroidism to have an approximate prevalence of 1.5-8% [10-13]. Primary hyperparathyroidism remains the most common cause of hypercalcemia-associated pancreatitis, whereas malignancy-related hypercalcemia is uncommon [10,11]. Hypercalcemia induces pancreatitis through premature activation of pancreatic enzymes and calcium-mediated ductal injury [10]. In MM, only isolated reports of hypercalcemia-induced pancreatitis have been documented, with no definitive incidence or prevalence data [12,13]. In our patient, markedly elevated serum calcium levels along with the absence of other etiologies strongly support hypercalcemia secondary to pPCL as the precipitating factor for pancreatitis, making the case presentation exceptionally rare.

The diagnosis of pPCL in the index case was established through peripheral smear and bone marrow examination, serum protein studies, and flow cytometry immunophenotyping. Peripheral blood smear revealed 51% circulating plasma cells, fulfilling the diagnostic criteria for PCL. Flow cytometry demonstrated loss of CD56 which is frequently associated with plasma cell detachment from the bone marrow microenvironment and contributes to leukemic and extra-medullary dissemination [3,7,9].

EMM represents an aggressive manifestation of PCD and is associated with poor prognosis [6-9]. PCL itself is considered a leukemic form of EMM because neoplastic plasma cells acquire the ability to survive independent of the bone marrow niche [7]. The presence of a scapular lytic lesion and systemic dissemination in our patient further supports the aggressive nature of the disease. EMM may occur either as bone-related EMM (bEMM) or soft tissue-related EMM (sEMM), with the latter carrying a worse prognosis due to hematogenous dissemination [6,18]. Studies by He et al. demonstrated significantly inferior outcomes in patients with soft tissue extra-medullary involvement compared with bone-related lesions [19].

Cytogenetic abnormalities play a major role in the pathogenesis and prognosis of pPCL and EMM. Our patient demonstrated 1q21 amplification on FISH analysis, a recognized high-risk cytogenetic abnormality associated with aggressive disease biology, increased proliferation, treatment resistance, and inferior survival [8]. Amplification of 1q21 promotes plasma cell survival through over-expression of genes such as *CKS1B* and *BCL9*, contributing to genomic instability and extra-medullary progression [8]. High-risk abnormalities including *TP53* deletion and del(1p32) have also been implicated in EMM pathogenesis [8].

Therapeutically, pPCL requires aggressive multimodality treatment. Novel induction regimens incorporating proteasome inhibitors, immunomodulatory agents, monoclonal antibodies, and corticosteroids have improved outcomes compared to conventional chemotherapy [18,20]. Our patient received induction with carfilzomib, lenalidomide, dexamethasone, and daratumumab followed by autologous stem cell transplantation, which currently remains one of the most effective consolidation strategies in eligible patients [18,20]. Subsequent consolidation with bortezomib, pomalidomide, and dexamethasone resulted in sustained clinical improvement after six cycles. Although the prognosis of pPCL remains poor overall, advances in targeted therapy, transplantation strategies, and minimal residual disease assessment are gradually improving survival outcomes [18,20].

## Conclusions

pPCL is a rare and aggressive PCD that may have a heterogeneous clinical presentation. Rarely, it may present as acute pancreatitis, secondary to hypercalcemia, even in younger patients. The atypical presentation, coupled with the young age at presentation, may be a potential cause of diagnostic dilemma. Early recognition of atypical presentations along with detailed laboratory evaluation, including peripheral smear examination, immunophenotyping, and cytogenetic analysis, helps in establishing a prompt diagnosis and initiating timely therapy.
